# Modulation des Darmmikrobioms zur Eradikation multiresistenter Erreger: Aktuelle Ansätze und Perspektiven

**DOI:** 10.1007/s00103-026-04229-3

**Published:** 2026-04-07

**Authors:** Tobias Weirauch, Maria J. G. T. Vehreschild

**Affiliations:** Universitätsklinikum Frankfurt, Medizinische Klinik II – Schwerpunkt Infektiologie, Goethe-Universität Frankfurt, Theodor-Stern-Kai 7, 60590 Frankfurt am Main, Deutschland

**Keywords:** Dekolonisierung, Mikrobiom, Nicht absorbierbare Antibiotika, Fäkaler Mikrobiota-Transfer, Probiotika, Decolonization, Microbiome, Non-absorbable antibiotics, Fecal microbiota transplantation, Probiotics

## Abstract

Die weltweite Zunahme von Antibiotikaresistenzen stellt eine der größten Herausforderungen für die moderne Medizin dar. Insbesondere die Besiedelung des Gastrointestinaltrakts mit multiresistenten Erregern gilt für verschiedene Gruppen als kritischer Risikofaktor für nosokomiale Infektionen. In diesem Zusammenhang rückt die gezielte Dekolonisation des Darms zunehmend in den Fokus klinischer Forschung. Nicht absorbierbare Antibiotika galten lange als vielversprechender Ansatz zur lokalen Eradikation – die mittlerweile generierte Evidenz spricht jedoch nicht für die Wirksamkeit dieses Ansatzes. Alternativverfahren wie der fäkale Mikrobiota-Transfer zeigen in Einzelfallberichten und kleinen Studien vielversprechende Ergebnisse bei der Dekolonisation multiresistenter Erreger. Auch lebende biotherapeutische Produkte (Live Biotherapeutic Products – LBPs) sowie bestimmte Probiotika werden als Optionen zur Modulation des Mikrobioms und zur Resistenzreduktion untersucht. Die Evidenzlage ist bislang heterogen und robuste randomisierte Studien fehlen größtenteils. Dieser Beitrag soll einen Überblick über den aktuellen Kenntnisstand zur gastrointestinalen Besiedelung mit multiresistenten Erregern geben. Darüber hinaus diskutiert er die klinische Relevanz nicht absorbierbarer Antibiotika bzw. den Stellenwert mikrobiombasierter Therapien im Kontext der globalen Antibiotikaresistenzkrise.

## Abstract (en)

The global rise in antibiotic resistance represents one of the greatest threats facing modern medicine. Colonization of the gastrointestinal tract with multidrug-resistant organisms is considered a critical risk factor for nosocomial infections across various patient populations. In this context, targeted decolonization strategies are moving into the focus of clinical research. For a long time, non-absorbable antibiotics were considered a promising approach for local eradication; however, the evidence generated on this question does not suggest sufficient clinical efficacy of this approach. Alternative strategies, such as fecal microbiota transplantation, have shown encouraging results in case reports and small-scale studies for the decolonization of multidrug-resistant organisms. Live biotherapeutic products and certain probiotics are also being explored as potential options for microbiome modulation and reduction of antimicrobial resistance. However, the current evidence base remains heterogeneous, and robust randomized controlled trials are largely lacking. This article aims to provide an overview on the current understanding of gastrointestinal colonization with multidrug-resistant organisms and to discuss the clinical relevance of non-absorbable antibiotics as well as the potential role of microbiome-based therapies in the context of the global antibiotic resistance crisis.

## Einleitung

Im Gastrointestinaltrakt findet sich eine komplexe und dynamische Vielfalt an kommensalen und potenziell pathogenen Mikroorganismen, die außerdem Antibiotikaresistenzgene beherbergen können [1]. Dabei ist hervorzuheben, dass auch eine physiologische Darmmikrobiota grundsätzlich Kommensalen enthält, die Resistenzgene tragen. Ob diese jedoch auch phänotypisch apparent werden und in der intestinalen Mikrobiota persistieren, hängt von verschiedenen Faktoren ab. Eine Schlüsselrolle spielt dabei die Häufigkeit, Dauer und Art vorhergehender Antibiotikaexpositionen. Daneben können auch Faktoren wie Ernährung und andere selektionswirksame Medikamente einen Einfluss nehmen [1, 2]. Innerhalb eines Wirtsorganismus kann es durch horizontale Gentransfermechanismen, wie Transformation, Transduktion oder bei Gram-negativen Bakterien insbesondere auch durch Konjugation, zu einer Übertragung von Resistenzgenen auf die unterschiedlichen Bakterien der intestinalen Mikrobiota kommen [3, 4]. Im Vergleich zu anderen Ökosystemen ist die Rate des Gentransfers im Gastrointestinaltrakt besonders hoch. Dies spielt insbesondere aus der One-Health-Perspektive eine maßgebliche Rolle, da Fäkalien in Abwässern als Hauptquelle der Übertragung antimikrobieller Resistenzgene zwischen verschiedenen Spezies identifiziert wurden [5]. Die Besiedlung des Gastrointestinaltraktes mit antibiotikaresistenten Bakterien erzeugt zwar selbst keine Symptome, stellt jedoch insbesondere bei Patientinnen und Patienten mit Grunderkrankungen, die deren Darmbarriere und/oder Immunkompetenz beeinträchtigen, einen erheblichen Risikofaktor für das Entstehen invasiver Infektionen dar.

Bevor wir uns der herausfordernden Dekolonisierung antibiotikaresistenter Erreger aus dem Gastrointestinaltrakt widmen, möchten wir die übergeordnete Bedeutung der Prävention ansprechen.

Eine der wichtigsten Säulen der Prävention stellen Antibiotic Stewardship (ABS) Programme dar. Ihre Bedeutung wird durch eine systematische Übersichtsarbeit mit Metaanalyse verdeutlicht, die zeigen konnte, dass ABS-Programme die Gesamtinzidenz von Infektionen und Kolonisation mit multiresistenten Gram-negativen Bakterien um 51 %, mit Extended-Spectrum-Betalaktamasen (ESBL) produzierenden Gram-negativen Bakterien um 48 % sowie mit Methicillin-resistentem *Staphylococcus aureus* (MRSA) um 37 % senken können [6]. Zahlreiche Studien konnten hohe Raten unangemessener Antibiotikaverordnungen dokumentieren, was die Notwendigkeit der verstärkten klinischen Integration von ABS-Programmen untermalt [7, 8]. Neben der präventiven Wirksamkeit konnten auch deutliche Einsparungen bei den Antibiotikakosten (−33,9 %) sowie eine Verkürzung der Krankenhausverweildauer (−8,9 %) mit verbesserten klinischen Ergebnissen nachgewiesen werden [9].

Vancomycin-resistente Enterokokken (VRE) und Carbapenem-resistente *Enterobacteriaceae* (CRE) haben sich mit rasanter Geschwindigkeit ausgebreitet und sind inzwischen in zahlreichen Regionen der Welt endemisch geworden [10, 11]. Die Erreger zeichnen sich oft durch die Fähigkeit einer langen asymptomatischen gastrointestinalen Besiedelung aus, ehe sie vor allem bei vulnerablen Patientinnen und Patienten (z. B. Immunsupprimierte und Organtransplantierte) zu Komplikationen durch invasive Infektionen führen. Ein erhöhtes Risiko in diesen Gruppen ergibt sich aus den häufigen Kontakten zu medizinischen Einrichtungen, häufigeren Interventionen sowie der erhöhten Antibiotikaexposition [12–14].

**Abb. 1:**
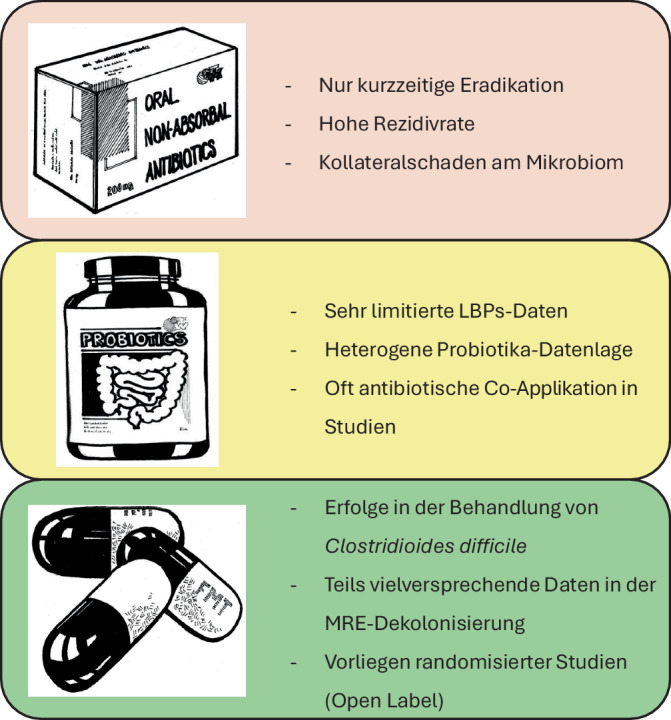
Vor- und Nachteile der diskutierten Therapieansätze in Stichpunkten. (*LBPs* Live Biotherapeutic Products, *MRE* multiresistente Erreger)

Da unter Wissenschaftlerinnen und Wissenschaftlern sowie Klinikerinnen und Klinikern zunehmend die Erkenntnis erwächst, dass ABS- und Infektionskontrollmaßnahmen allein die Ausbreitung antimikrobieller Resistenzen nicht im erwünschten Maße eindämmen können, gewinnen alternative Ansätze an Bedeutung. Die Rolle der Darmmikrobiota rückt dabei immer stärker in den Fokus der Forschung. Ihre Komposition spielt neben exogenen Faktoren eine zentrale Rolle für die Persistenz von multiresistenten Erregern und könnte somit potenziell zur Eindämmung von Resistenzen beitragen [15]. Infolgedessen wurden verschiedene Strategien zur Wiederherstellung einer gesunden Darmmikrobiota entwickelt. Erste Versuche wurden dabei bereits mit dem fäkalen Mikrobiota-Transfer (FMT), sog. Live Biotherapeutic Products (LBPs), Probiotika und Bakteriophagenapplikationen umgesetzt. Die gegenwärtig zur Verfügung stehenden Daten reichen aber noch nicht aus, um richtungsweisende Behandlungsempfehlungen auszusprechen. Dieser Artikel soll den Leserinnen und Lesern einen Überblick über potenzielle Methoden zur Dekolonisierung multiresistenter Erreger (MRE) im Gastrointestinaltrakt geben. Dabei stehen nicht absorbierbare Antibiotika, der FMT, LBPs und Probiotika im Vordergrund, deren Vor- und Nachteile in Abb. 1 zusammengefasst werden. Das Potenzial der Bakteriophagen wird in einem gesonderten Artikel thematisiert.

## Nicht absorbierbare Antibiotika

Seit vielen Jahrzehnten werden orale nicht resorbierbare Antibiotika als potenzielle Dekolonisationsoptionen für antibiotikaresistente Bakterien diskutiert, da ihre Wirkung in der Regel auf den Gastrointestinaltrakt beschränkt ist und somit systemische Nebenwirkungen seltener auftreten [16]. Dazu gehört auch der Ansatz der selektiven Darmdekontamination (Selective Digestive Decontamination – SDD). Allerdings beeinflussen nicht resorbierbare Antibiotika aufgrund ihres breiten Spektrums nicht nur das bakterielle Wachstum der unerwünschten Besiedler, sondern auch die physiologische Zusammensetzung des intestinalen Mikrobioms [17]. Die Auswirkungen dieser Modifikation sind im Einzelfall nur schwer vorherzusagen, wobei sie zu schwerwiegenden Komplikationen, wie beispielsweise einer *Clostridioides-difficile*-Infektion (CDI) mit pseudomembranöser Kolitis oder einem toxischen Megakolon, führen können. In anderen Fällen kann bei Ko-Existenz von zwei oder mehreren Resistenzgenen auf einem Plasmid (z. B. Resistenzgene gegen Aminoglykoside und Carbapeneme) eine antibiotische Therapie zur sogenannten Ko-Selektion resistenzgentragender Plasmide führen, wenngleich nur eine Antibiotikaklasse eingesetzt wurde [18].

Berücksichtigt man zudem die Erkenntnisse aus zurückliegenden Studien (VRE, CRE, ESBL), die den nicht absorbierbaren Antibiotika nur kurzfristige Effekte attestierten, ergibt sich hieraus zumindest zur Dekolonisation keine überzeugende Behandlungsoption [19–22]. Diese Position wird auch durch die klinischen Leitlinien der ESCMID-EUCIC (European Society of Clinical Microbiology and Infectious Diseases – ESCMID; European Committee on Infection Control – EUCIC) aus 2019 unterstützt, die von einer generellen Empfehlung zur Dekolonisation multiresistenter Gram-negativer Bakterien absieht [9].

## Fäkaler Mikrobiota-Transfer

Beim FMT wird die Mikrobiota gesunder, sorgfältig gescreenter Spender mittels rektaler Einläufe, nasogastraler bzw. duodenaler Sonden, koloskopischer Applikation oder verkapselter Präparate in den Darm der Empfänger übertragen. Dabei sollen die Diversität und Funktion der kommensalen Mikrobiota verbessert werden, was zu einer optimierten Regulation verschiedener Organsysteme und einer sog. Kolonisationsresistenz gegenüber potenziellen Pathogenen führen kann [23, 24]. Vermutlich reicht der Ansatz des FMT bis ins 4. Jahrhundert zurück, wo chinesische Medizinerinnen und Mediziner bereits erste Stuhlübertragungen dokumentierten. Einzug in die wissenschaftliche Literatur fand dieser Ansatz durch Ben Eiseman in den späten 1950er-Jahren. Schon damals konnte er sich die Methode erfolgreich in der Therapie der pseudomembranösen Enterokolitis zunutze machen. Fast 70 Jahre später gilt der FMT als Goldstandard in der Therapie der rezidivierenden *Clostridioides-difficile*-Infektion.

Während einige Patientinnen und Patienten zeitweise über Nebenwirkungen wie Diarrhö, Übelkeit, abdominelle Schmerzen, subfebrile Temperaturen, Blähungen und Obstipation berichten, sind schwere unerwünschte Ereignisse unter Nutzung eines umfassenden Spenderscreenings selten. Letzteres ist essenziell, um den Ausschluss von: i) pathogenen Mikroorganismen (Bakterien, Viren, Parasiten, Pilze), ii) schweren Komorbiditäten, iii) bestimmten Vorbehandlungen (Antibiotika, Immunsuppressiva, Protonenpumpenhemmer (PPI), Krankenhausaufenthalte), iv) Risikoreisen sowie v) sozialen Risikofaktoren (z. B. Drogenkonsum, sexuelles Risikoverhalten) sicherzustellen [25]. Einige Risiken, wie Aspirationspneumonien, stehen zudem mit der Verabreichungsform in Zusammenhang. Der Applikationsweg des FMT über den oberen Gastrointestinaltrakt ist mit höheren Nebenwirkungsraten (43,9 %) assoziiert als die Verabreichung über den unteren Gastrointestinaltrakt (17,7 %) oder per Kapsel [26].

Aufgrund des Erfolges in der Behandlung von *Clostridioides-difficile*-Infektionen, mit signifikant höheren Ansprechraten unter FMT (71 %) im Vergleich zu Fidaxomicin (33 %) oder Vancomycin (19 %; [27]), wird der FMT auch als potenzielle Strategie zur Dekolonisation von Patientinnen und Patienten, die intestinal mit multiresistenten Bakterien kolonisiert sind, diskutiert. Beispielsweise zeigte ein Review aus dem Jahr 2019 eine kumulative Dekolonisationsrate von 68,2 % (30/44) bei Gram-positiven Bakterien (MRSA, VRE) und 70,6 % (72/102) bei Gram-negativen Erregern (z. B. Carbapenemase bildende *Klebsiella pneumoniae*, CRE oder ESBL-*Escherichia coli*), jedoch variierte die Wirksamkeit unter den Erregerklassen [28]. Ein systematisches Review aus dem Jahr 2022, dessen Fokus auf CRE (insbesondere *Klebsiella pneumoniae* und *Escherichia coli*) lag, konnte eine Dekolonisationsrate von 78,7 % nach sechs bis zwölf Monaten bei 112 FMT-Empfängerinnen und -Empfänger zeigen [29]. Der klinische Nutzen des FMT kann bei verschiedenen multiresistenten Darmbakterien noch nicht abschließend bewertet werden. Hoffnung geben allerdings neben einzelnen klinischen Studien auch molekulargenetische Realtime-PCR-Untersuchungen, die zur Detektion der Expression von Antibiotikaresistenzgenen herangezogen wurden. Darin zeichnet sich nach durchgeführtem FMT eine verminderte Expression von bakteriellen Resistenzgenen im Stuhl ab, was zwar nicht der Dekolonisation des Bakteriums, jedoch der multiresistenten Eigenschaft entsprechen könnte [30]. Am Beispiel des Resistenzgens VanA, das eine Modifikation der bakteriellen Polypeptidketten bedingt, kann die FMT-bedingte VanA-Expressionsreduktion die Bindung von Glykopeptid-Antibiotika (z. B. Vancomycin oder Teicoplanin) an bakterielle Polypeptidketten verbessern.

Weitere randomisierte kontrollierte Studien sind notwendig (Randomized Controlled Trials – RCT), um den Nutzen des FMT pathogenspezifisch zu validieren. Zwei derartige Studien befinden sich derzeit bereits in Arbeit [31, 32]. Es wird jedoch noch weitere Bemühungen brauchen, bis die Real-World Effectiveness des FMT in diesem Zusammenhang beurteilt werden kann.

Da die regulatorische Bewertung des FMT international sehr unterschiedlich ausfällt, variiert die Verfügbarkeit stark. Zudem beeinflussen die Kosten des umfangreichen Spenderscreenings die Ausdehnung der Nutzung [25]. Eine dänische Studie zeigte hingegen, dass eine Reduktion der Therapiekosten bei rezidivierender CDI um 42 % durch den FMT möglich ist [33]. Eine aktuelle Übersicht bestätigt die Kosteneffizienz des FMT hinsichtlich Behandlungsaufwand und Krankenhausverweildauer im Vergleich zu konventionellen antibiotischen Strategien [34]. Inwieweit sich die ökonomischen Aspekte auf die Dekolonisation von antibiotikaresistenten Darmbakterien auswirkt, kann gegenwärtig nur schwer eingeschätzt werden.

## Live Biotherapeutic Products, Probiotika und Präbiotika

LBPs werden von der US-amerikanischen Food and Drug Administration (FDA) und der Europäischen Arzneimittel-Agentur (EMA) als ein biologisches Produkt definiert, das 1) lebende Organismen, etwa Bakterien, enthält, 2) zur Prävention, Behandlung oder Heilung von Krankheiten oder Beschwerden beim Menschen eingesetzt wird und 3) kein Impfstoff ist.

Probiotika werden von der International Scientific Association for Probiotics and Prebiotics (ISAPP) als lebende Mikroorganismen definiert, die, in ausreichender Menge verabreicht, einen gesundheitlichen Nutzen für den Wirt erbringen können [35]. Sie enthalten einen oder mehrere Stämme von Mikroorganismen, z. B. *Lactobacillus, Escherichia coli*, Enterokokken oder Hefen, und werden nach Gattung, Art, ggf. Unterart sowie einer alphanumerischen Stammbezeichnung benannt. Die FDA und die Europäische Behörde für Lebensmittelsicherheit (EFSA) stufen Probiotika aktuell als Nahrungsergänzungsmittel ein. Tab. 1 fasst die wichtigsten Differenzierungskriterien zwischen LBPs und Probiotika zusammen.

**Tab. 1:** Differenzierung zwischen Live Biotherapeutic Products und Probiotika.

	Live Biotherapeutic Products	Probiotika
*Definition*	Arzneimittel, die lebende Mikroorganismen enthalten und zur Prävention, Behandlung oder Heilung von Krankheiten bestimmt sind	Lebende Mikroorganismen, die bei ausreichender Aufnahme gesundheitsfördernde Wirkungen entfalten, jedoch nicht als Arzneimittel eingestuft werden
*Regulatorische Aspekte*	Sie unterliegen der Regulierung als biologische Arzneimittel gemäß Arzneimittelgesetzgebung und klinischen Prüfverordnungen	Sie werden als Lebensmittel oder Nahrungsergänzungsmittel klassifiziert und unterliegen allgemeinen Vorschriften zur Lebensmittelsicherheit und gesundheitsbezogenen Angaben
*Genehmigungsprozess*	Erfordern präklinische Studien, klinische Prüfungen sowie eine Zulassung durch zuständige Aufsichtsbehörden (z. B. EMA, FDA)	Unterliegen lebensmittelrechtlichen Sicherheitsanforderungen; klinische Studien sind in der Regel nicht erforderlich, es sei denn, es wird eine gesundheitsbezogene Angabe gemacht
*Wirkmechanismus*	Weisen klar definierte Wirkmechanismen auf, die häufig durch klinische Evidenz für eine bestimmte Erkrankung gestützt sind	Die gesundheitsfördernden Effekte sind allgemein gehalten, etwa zur Unterstützung des Mikrobioms, ohne krankheitsspezifische Wirksamkeitsansprüche
*Herstellungsstandards*	Müssen den Anforderungen der Guten Herstellungspraxis (GMP) für Arzneimittel entsprechen	Die Herstellung erfolgt typischerweise nach lebensmittelrechtlichen Standards
*Exemplarische Anwendungsbereiche*	Infektionen mit *Clostridioides difficile, *chronisch entzündliche Darmerkrankungen und weitere Indikationen	Unterstützung der Verdauungsgesundheit, Stärkung des Immunsystems und Wiederherstellung der Darmflora nach Antibiotikatherapie

*EMA* Europäische Arzneimittel-Agentur, *FDA* Food and Drug Administration

Die Wirkmechanismen von LBPs und Probiotika beruhen vor allem auf Interaktion mit dem Darmmikrobiom und dem Wirt. Sie entfalten ihre Wirkung durch die Modulation der Zusammensetzung und metabolischen Aktivität der intestinalen Mikrobiota, die kompetitive Verdrängung von Pathogenen sowie die Produktion antimikrobieller Substanzen. Darüber hinaus stärken sie die intestinale Barrierefunktion durch die Hochregulation von Tight-Junction-Proteinen und beeinflussen das angeborene sowie adaptive Immunsystem [36, 37].

Dem Einsatz von LBPs in der Rezidivprävention von *Clostridioides-difficile*-Infektionen konnte eine randomisierte, placebokontrollierte Studie positive Effekte zuschreiben [38]. Trotz des hohen klinischen Bedarfs finden sich bislang weder Studien noch nennenswerte Bestrebungen, die den Einsatz von LBPs zur gezielten Dekolonisierung von MRE untersuchen. Angesichts der zunehmenden MRE-Prävalenz und der limitierten therapeutischen Optionen sollten klinische Entwicklungen in diesem Bereich unterstützt werden.

Betrachtet man die Literatur über Dekolonisationsversuche Gram-negativer Bakterien mithilfe von Probiotika, zeigte *Escherichia coli* Nissle 1917 in einer placebokontrollierten Studie keine Wirkung auf die Dekolonisation von Norfloxacin-resistenten *E. coli* im Stuhl [39]. Zwei kleine Studien zur Anwendung von Probiotika bei ESBL/CRE zeigten widersprüchliche Ergebnisse: Eine Untersuchung mit acht probiotischen Bakterienstämmen zeigte keinen signifikanten Effekt [40]. Choi et al. waren hingegen mit einem Protokoll aus *Bacillus subtilis* und *Enterococcus faecalis* zur CRE-Dekolonisation bei 9 von 13 Patientinnen und Patienten (69,2 %) erfolgreich [41]. Die Studie verfügte jedoch über keine Kontrollgruppe. Ljungquist et al. evaluierten Vivomixx® in einer kleinen RCT zur ESBL-Dekolonisation. Nach einem Jahr waren 12,5 % (5/40) der Probiotikagruppe und 5 % (2/40) der Kontrollgruppe dekolonisiert [40].

Blickt man auf die Dekolonisationsversuche, die Bakterien des Gram-positiven Spektrums adressierten, zeigten die Arbeiten mit den höchsten Gütekriterien (doppelblind, randomisiert, placebokontrolliert) zur VRE-Dekolonisation mit *Lactobacillus rhamnosus GG* keinen signifikanten Effekt [42, 43]. Demgegenüber steht unter anderem eine kleine Studie von Manley et al., bei der bei 11/11 Patientinnen und Patienten eine erfolgreiche VRE-Elimination nach dreiwöchiger Behandlung mit *L. rhamnosus GG* dokumentiert wurde [44]. Eine Folgestudie an Neugeborenen zeigte zudem eine Dekolonisation bei 21/22 Patientinnen und Patienten innerhalb von sechs Monaten [45]. Eine thailändische Phase-II-Studie (doppelblind, randomisiert, placebokontrolliert) konnte zudem nach einer 30-tägigen Einnahme von *Bacillus subtilis* eine signifikante Reduktion von *Staphylococcus aureus* im Stuhl und in der Nase zeigen [46]. Weitere Studien und Fallserien liefern inkonsistente Ergebnisse [10, 47, 48]. Darüber hinaus gibt es ebenfalls keine Evidenz dafür, dass Probiotika die Transmission von VRE oder antibiotikaresistenten Gram-negativen Bakterien verhindern könnten [49, 50].

Insgesamt zeigen randomisierte, kontrollierte Studien, dass Probiotika in der Regel sicher sind [51]. Ihre Wirksamkeit oder Nebenwirkungen lassen sich jedoch aufgrund heterogener Protokolle bzw. der begleitenden Applikation von Antibiotika schwer abschätzen. Ob die teils kostspieligen Präparate in der Dekolonisation von antibiotikaresistenten Darmbakterien oder anderen Anwendungsbereichen eine Alternative werden, muss zunächst durch weitere Studien geklärt werden. Aktuell gibt es für viele medial genannte Versprechungen keine oder nur unzureichende wissenschaftliche Belege.

Neben der Nutzung von Bakterien selbst kann über den Einsatz von Präbiotika das Mikrobiom ebenfalls beeinflusst werden. Dabei handelt es sich um nicht verdaubare Nahrungsbestandteile, die selektiv das Wachstum nützlicher Darmbakterien fördern sollen. Frühere Studien zeigten ein Wachstum von *Lactobacillus*- und *Bifidobacterium*-Stämmen unter Präbiotikagabe [52]. *In vitro* konnte zudem gezeigt werden, dass bestimmte Präbiotika das Wachstum und die antibakterielle sowie Antibiofilm-Aktivität von *L. rhamnosus* fördern [53]. Weitere *in vitro *Arbeiten konnten die Wachstumsinhibition potenziell pathogener Bakterien wie *Klebsiella* und *Enterobacter* spp. durch präbiotische Fasern zeigen [54]. Dabei wurden jedoch keine multiresistenten Bakterienstämme untersucht. Die Übertragbarkeit auf *in vivo *Versuche bleibt unklar.

Im Tiermodell (Maus) konnte hingegen eine Kombination aus Inulin und Pantoprazol untersucht werden, die zu einer signifikanten Reduktion von ESBL-*Escherichia-coli* im Stuhl führte [55]. Eine weitere Arbeit am Tiermodell zeigte wiederum, dass Fucoidane die Dekolonisation von *Pseudomonas aeruginosa* erleichterten und zudem einen Rückgang von *Enterobacteriaceae *und *Enterococcaceae* begünstigten [56]. Diese Daten deuten auf eine Bindungshemmung virulenter Faktoren an Mucinen hin [56]. Ob und inwieweit diese Aussagen auf das humane Mikrobiom übertragbar sind, bleibt jedoch unklar. Zahlreiche Limitationen sollten bei der Interpretation und Ableitungsversuchen berücksichtigt werden. Zudem handelte es sich um präventive Anwendungen und nicht um Eradikationsversuche. Diese Effekte lassen sich möglicherweise unterstützend zur Dekolonisation von antibiotikaresistenten Bakterien nutzen. Allerdings existieren kaum klinische Studien, die den Effekt von Präbiotika in diesem Zusammenhang untersuchen.

## Fazit

Um der komplexen globalen Resistenzkrise entschlossen entgegenzutreten, müssen weitreichende Veränderungen angestrebt werden. Neben einer rationalen Antibiotikaverordnung gilt es, die Möglichkeiten der nichtantibiotischen Präventions- und Eradikationsstrategien besser zu verstehen. Bei der Vielzahl von multiresistenten Erregern bzw. den komplexen Resistenzmechanismen werden individualisierte Therapiekombinationen den standardisierten Formulierungen sehr wahrscheinlich überlegen sein. Gegenwärtig können wir jedoch keine erregerspezifischen, evidenzbasierten Grundsätze formulieren, wobei das Potenzial der verschiedenen mikrobiota- und ernährungsbasierten Dekolonisationsansätze noch nicht vollständig erschlossen wurde. Angesichts der unzureichenden therapeutischen Optionen sind jedoch erhebliche Investitionen in die Forschung unerlässlich, um ein besseres Verständnis der nichtantibiotischen Behandlungsoptionen zu generieren.

## Data Availability

Nicht zutreffend. Bei diesem Artikel handelt es sich um eine narrative Übersichtsarbeit, die auf bereits veröffentlichten Studien basiert. Es wurden im Rahmen dieser Arbeit keine neuen Daten erstellt oder analysiert.
